# TyG Index Performs Better Than HOMA-IR in Chinese Type 2 Diabetes Mellitus with a BMI < 35 kg/m^2^: A Hyperglycemic Clamp Validated Study

**DOI:** 10.3390/medicina58070876

**Published:** 2022-06-30

**Authors:** Ping Luo, Yaoquan Cao, Pengzhou Li, Weizheng Li, Zhi Song, Zhibing Fu, Hui Zhou, Xianhao Yi, Liyong Zhu, Shaihong Zhu

**Affiliations:** Department of General Surgery, Third Xiangya Hospital, Central South University, Changsha 410013, China; luoping@csu.edu.cn (P.L.); yaoquan@csu.edu.cn (Y.C.); 602223@csu.edu.cn (P.L.); 2204060326@csu.edu.cn (W.L.); songzhi200@csu.edu.cn (Z.S.); fzb123@csu.edu.cn (Z.F.); 208302054@csu.edu.cn (H.Z.); 198302057@csu.edu.cn (X.Y.)

**Keywords:** type 2 diabetes mellitus, insulin resistance, hyper-insulinemic euglycemic clamp, TyG index, HOMA-IR

## Abstract

*Background and objectives*: Chinese type 2 diabetes mellitus (T2DM) patients are characterized by a low body mass index (BMI), and significant insulin resistance (IR). The triglyceride glucose (TyG) index has not been studied as a means of assessing IR in Chinese T2DM patients with a BMI < 35 kg/m^2^. *Materials and Methods*: An open-label cross-sectional study recruited 102 Chinese T2DM patients with a BMI < 35 kg/m^2^. The hyper-insulinemic euglycemic clamp, homeostatic model assessment of IR (HOMA-IR), and TyG index were used to determine the level of IR. Based on Pearson’s correlations, glucose disposal rate (GDR), TyG index, and HOMA-IR were analyzed. HOMA-IR and TyG index for IR were evaluated using multiple linear regression and multivariate logistic regression analyses. On the basis of the receiver operating characteristic (ROC) curve, the sensitivity, specificity, and optimal cut-off value of HOMA-IR and the TyG index were determined. *Results*: The mean values of GDR, HOMA-IR, and TyG index were 4.25 ± 1.81, 8.05 ± 7.98, and 8.12 ± 0.86 mg/kg/min, respectively. Pearson’s correlation coefficient was −0.418 between GDR and TyG index and −0.324 between GDR and HOMA-IR. ROC curve analysis showed that, among both sexes, the TyG index was a better discriminator of IR than HOMA-IR. The area under the ROC curve (AUC) of the TyG index (0.785, 0.691–0.879) was higher than that of HOMA-IR (0.73, 0.588–0.873) in all genders. The optimal cut-off values of the TyG index and HOMA-IR were 7.99 and 3.39, respectively. *Conclusions*: The TyG index showed more effectiveness in identifying IR in Chinese T2DM patients with a BMI < 35 kg/m^2^ compared to HOMA-IR.

## 1. Introduction

The incidence of T2DM has increased dramatically across the globe [1]. Diabetes and related comorbidities were the leading cause of death in China in 2019 [2]. Chinese T2DM patients are characterized by a relatively young age, a low BMI, and significant IR [3,4]. It is well documented that the two major pathological features of T2DM are IR and β-cell dysfunction [5,6]. The incidence of T2DM is related more to IR than to β-cell dysfunction in Chinese adults [7]. Therefore, it is very important to find a simple and reliable index to assess the IR of patients with T2DM.

HOMA-IR, based on fasting glucose and insulin, is the most popular index for evaluating IR in diabetes populations [8]. Unfortunately, HOMA-IR is the least accurate and varies partly due to the lack of standardization of insulin immunoassays [9]. Kang et al. reported that HOMA-IR had limitations to evaluate IR in low BMI T2DM patients with β-cell malfunction and insulin secretory defects [10]. A hyper-insulinemic euglycemic clamp is considered the gold standard to assess IR, but its disadvantages are obvious and fatal because it is laborious, complex, and expensive [11]. The TyG index, an emerging and reliable indicator, has been used to identify IR in different populations and diseases [12,13,14].

To the best of our knowledge, the value of the TyG index needed to assess IR in Chinese T2DM patients with a low BMI has not been studied. Therefore, we prospectively evaluated the potential of using the TyG index to assess IR in Chinese patients with T2DM and a BMI < 35 kg/m^2^.

## 2. Materials and Methods

### 2.1. Study Population

**Figure 1:**
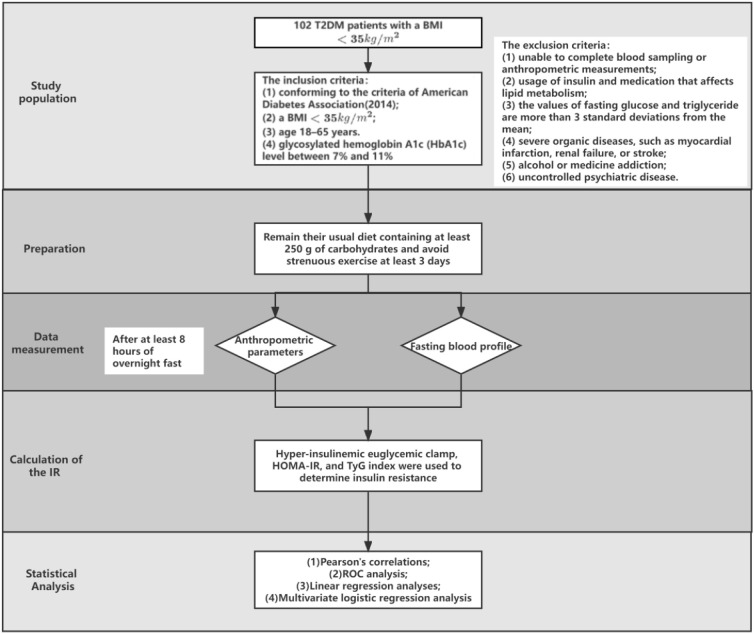
Schematic of the research methods.

A cross-sectional study was conducted in the Department of General Surgery, Third Xiangya Hospital, Central South University (Changsha, China). One hundred and two T2DM patients with a BMI < 35 kg/m^2^ were prospectively consecutively recruited. The detailed steps for the search are presented in Figure 1. With the approval of the protocol by the Ethical Committee of our hospital (R19025), informed consent was signed before the study.

The inclusion criteria were as follows: (1) Conforming to the criteria of American Diabetes Association (2014) [15]; (2) a BMI < 35 kg/m^2^; (3) aged 18–65 years; (4) glycosylated hemoglobin A1c (HbA1c) level between 7% and 11%. The exclusion criteria were: (1) Unable to complete blood sampling or anthropometric measurements; (2) usage of insulin and medication that affects lipid metabolism; (3) the values of fasting glucose and triglyceride are more than three standard deviations from the mean; (4) severe organic diseases, such as myocardial infarction, renal failure, or stroke; (5) alcohol or medicine addiction; and (6) uncontrolled psychiatric disease.

### 2.2. Study Protocol

All participants were required to maintain their usual diet containing at least 250 g of carbohydrates and avoid strenuous exercise at least 3 days before the procedure. Medical treatment of T2DM including sulfonylureas and/or biguanide was required to continue as usual before the study.

### 2.3. Assays

Anthropometric parameters and fasting blood profile were performed after at least 8 h of overnight fast. Plasma glucose was determined using the glucose oxidase method (Beckman Coulter Inc, Brea, CA, America). Triglycerides were enzymatically analyzed by using the spectrophotometric method (Beckman Coulter Inc, Brea, CA, America). Plasma insulin was detected by radioimmunoassay (Roche, Basel, Switzerland). HbA1c was analyzed by using a high-performance liquid chromatograph (Hitachi, Tokyo, Japan). The level of IR was evaluated by a hyper-insulinemic euglycemic clamp, HOMA-IR, and the TyG index. The TyG index was calculated as ln [fasting triglyceride (mg/dL) × fasting glucose (mg/dL)/2] [16]. HOMA-IR was calculated as fasting glucose (mmol/L) × fasting insulin (mU/mL)/22.5 [8].

The operation procedure of the hyper-insulinemic euglycemic clamp was performed as in our previous study [17]. A clamping test was performed after a 12 h overnight fast. Catheters were inserted into the antecubital and dorsal vein for infusion and blood sampling, respectively. Insulin (Humulin R, Eli Lilly, Indianapolis, IN, USA) was infused at a constant rate of 40 mU/kg/min for 150 min. Serum glucose level was measured every 5 min with a glucose analyzer. Dextrose 20% was administered intravenously at variable rates to maintain a steady glucose level of 5.0 mmol/L. Glucose disposal rate (GDR) was calculated at steady-state intervals.

### 2.4. Statistical Analysis

The data analysis was performed using SPSS software version 26 (SPSS Inc. Chicago, USA). Continuous variables are expressed as mean ± standard deviation. Categorical variables are expressed as frequencies and percentages. Pearson’s correlations between GDR, TyG index, and HOMA-IR were analyzed. The sensitivity, specificity, and the optimal cut-off value of the TyG index and HOMR-IR to evaluate IR were calculated using an ROC curve. The AUC, as a criterion of diagnostic accuracy, was evaluated. After GDR was log-transformed (natural logarithm) to approximate normal distributions, linear regression analyses with log GDR as the dependent variable were conducted to assess associations between TyG as well as HOMA-IR with GDR. The predicted value for each index was calculated as the R^2^ of the entire regression model minus the R^2^ of the underlying model excluding each index. The GDR was divided based on the quintile into grades I–III before the multivariate logistic regression analysis was performed to calculate the odds ratio and 95% confidence interval (CI) for IR. Regression analysis took gender specificity into account. Statistical significance was defined as *p* values < 0.05.

## 3. Results

A total of 102 T2DM patients with a BMI < 35 kg/m^2^ were enrolled. According to the exclusion criteria (3), four patients were excluded. The average age and BMI were 42.34 ± 12.86 years and 29.20 ± 3.52 kg/m^2^, respectively. The mean duration of T2DM was 5.84 ± 4.33 years. Sixty-six subjects were treated with metformin to control blood glucose. The mean duration of metformin was 4.16 ± 4.57 years. Totals of 53.1 and 50 percent of patients had hypertension and nonalcoholic fatty liver disease, respectively.

The average fasting glucose and triglyceride were 167.09 ± 63.39 mg/dL and 56.29 ± 53.71 mg/dL, respectively. The mean values of GDR, HOMA-IR, and TyG index were 4.25 ± 1.81, 8.05 ± 7.98, and 8.12 ± 0.86 mg/kg/min, respectively. The clinical characteristics of subjects are shown in Table 1. All clinical characteristics did not show significant differences between both sexes.

**Figure 2:**
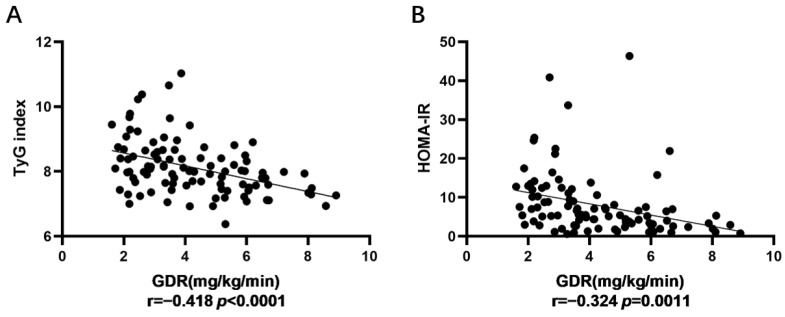
(**A**): Correlations between TyG index and GDR; (**B**): correlations between HOMA-IR and GDR.

All of the following analyses were stratified by sex because of significant differences in lipid profiles between men and women [18]. As shown in Table 2 and Figure 2, the Pearson’s correlations coefficient between GDR and the TyG index was −0.418 (−0.412 in men and −0.431 in women). On the other hand, the correlation between GDR and HOMA-IR was −0.324 (−0.291 in men and −0.364 in women). Compared with HOMA-IR, the TyG index showed a better performance in identifying diabetes patients with IR in both sexes.

As shown in Table 3, both the TyG index and HOMA-IR made a significant incremental additive contribution to the prediction of IR in both sexes. However, the additional percentage of variation in IR explained by the TyG index was stronger than by HOMA-IR in men (0.134 versus 0.044) and women (0.81 versus 0.003). The direct comparative ORs and 95% CIs for the TyG index and HOMA-IR are presented in Table 4. Compared with HOMA-IR (1.08 in men and 1.11 in women), among both sexes, the OR for IR was highest in the TyG index (2.22 in men and 3.07 in women).

**Figure 3:**
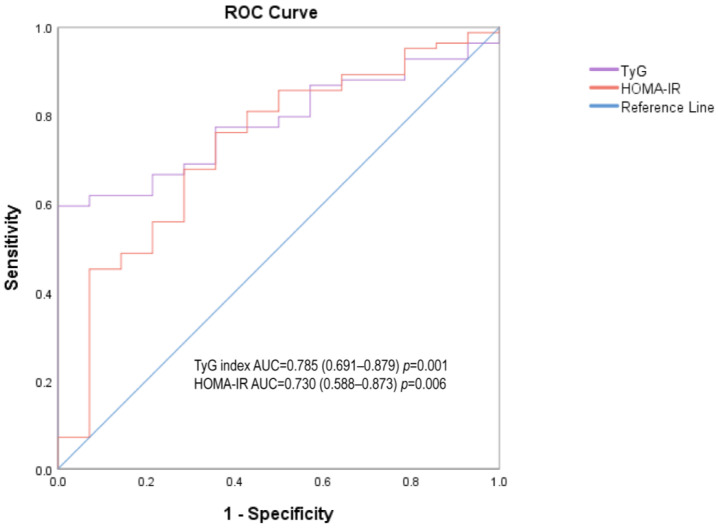
Receiver operating characteristic (ROC) analysis for TyG index and HOMA-IR in the identification of insulin resistance.

As shown in Table 5 and Figure 3, the AUC of the TyG index (0.785, 0.691–0.879) was higher than that of HOMA-IR (0.73, 0.588–0.873) among both sexes, as well as in men (0.774 versus 0.709) and women (0.794 versus 0.769). The AUC analysis indicated that the TyG index was a better surrogate maker to evaluate IR than HOMA-IR. The optimal cut-off values of the TyG index and HOMA-IR were 7.99 and 3.39 with a sensitivity of 59.5% and 76.2% and a specificity of 100% and 64.3% respectively.

## 4. Discussion

T2DM is characterized by impaired glucose and lipid metabolism associated with IR [6,19]. There is strong evidence that IR is closely linked to the pathophysiology of T2DM [20,21]. It is vital to identify IR with a reliable and simple indicator.

HOMA-IR, the TyG index, and a hyper-insulinemic euglycemic clamp were used in our study to investigate IR in T2DM patients with a BMI < 35 kg/m^2^. Our results indicate that the TyG index can be used to identify IR among Chinese adults with T2DM who have a low BMI, as it closely matches the hyper-insulinemic euglycemic clamp. HOMA-IR was a validated and widely used method for identifying IR, but laboratory measurements of plasma insulin have not been standardized [22]. Moreover, a study has shown that the majority of commercial assays lack precision and cross-reactivity [9]. According to Muniyappa et al. [23], HOMA-IR has no linear correlation with the clamp test. Similar results were found in a Korean population with T2DM and a lower BMI [10]. HOMA-IR and GDR showed a correlation based on our results, but it was lower than the correlation for the TyG index in both sexes. Meanwhile, multiple linear regression showed that the additional percentage of variation in IR explained by the TyG index is stronger than by HOMA-IR in all subjects. In addition, it indicated that the TyG index may be a better maker to assess IR than HOMA-IR.

This simple lipid-glucose index, based on fasting triglyceride and glucose levels, is affordable and readily available in most clinical laboratories [13,24]. The TyG index was demonstrated to be a simple and reliable measure of IR by Guerrero-Romero et al. [12]. For assessing IR in the Brazilian population, Vasques et al. showed that the TyG index outperformed HOMA-IR [11]. In our study, Pearson’s correlation coefficient between the TyG index and GDR (−0.418) was lower than that of HOMA-IR (−0.324) in all genders. Multivariate logistic regression showed that the value of OR in the TyG index was higher than that of HOMA-IR. In ROC analysis, the AUC of the TyG index (0.785, 0.691–0.879) was higher than that of HOMA-IR (0.73, 0.588–0.873), supporting that the TyG index was a better indicator than HOMA-IR in identifying IR in Chinese T2DM patients with a BMI < 35 kg/m^2^.

The main advantage of this study was comparing the gold standard clamp test with the TyG index and HOMA-IR. Using the TyG index, we also calculated the optimal cut-off value for IR identification in our study population. The present study is without a doubt limited. To begin with, the sample size was small. Additionally, we did not take into account the variability in fasting triglyceride levels when calculating the TyG index.

## 5. Conclusions

The TyG index can be regarded as an accurate and reliable indicator of IR and outperformed HOMA-IR in Chinese T2DM patients with a BMI < 35 kg/m^2^.

## Figures and Tables

**Table 1:** Clinical characteristics of patients (*n* = 98).

Characteristics	Total (*n* = 98)		Men (*n* = 57)		Women (*n* = 41)		*p* Value
	Mean ± SD	Median (Min, Max)	Mean ± SD	Median (Min, Max)	Mean ± SD	Median (Min, Max)	
Age (years)	42.34 ± 12.86	42 (18, 67)	42.46 ± 12.43	43 (18, 64)	42.17 ± 13.58	41 (18, 67)	0.914
Body mass index (kg/m^2^)	29.20 ± 3.52	29.75 (21.88, 34.95)	28.94 ± 3.58	28.72 (21.88, 34.77)	29.56 ± 3.46	29.95 (22.19, 34.95)	0.394
Waist circumference (cm)	99.92 ± 9.88	98 (61, 131)	99.93 ± 10.52	97.5 (61, 131)	99.92 ± 9.03	99 (80, 118)	0.994
Duration of T2DM (years)	5.84 ± 4.33	5 (1, 15)	6.07 ± 4.18	5 (1, 15)	5.51 ± 4.55	4 (1, 15)	0.532
Metformin (n)	66 (67.35%)		37 (64.91%)		29 (70.73%)		0.549
Duration of metformin (years)	4.16 ± 4.57	2 (1, 15)	4.04 ± 4.51	2 (1, 15)	4.34 ± 4.69	2 (1, 15)	0.745
Fasting glucose (mg/dL)	167.09 ± 63.39	155.88 (68.04, 392.04)	161.22 ± 62.88	151.2 (68.04, 392.04)	175.25 ± 63.97	165.06 (83.7, 297.72)	0.282
HbA1c (%) HbA1c (mmol/mol)	8.50 ± 1.87 62.65 ± 17.79	8.3 (5.6, 16.8) 59.56 (37.71, 106.56)	8.55 ± 1.58 68.33 ± 17.77	8.3 (6.2, 12.3) 66.12 (44.26, 110.93)	8.44 ± 2.24 68.79 ± 24.48	8.1 (5.6, 16.8) 65.03 (37.71, 160.11)	0.793 0.503
Triglyceride (mg/dL)	56.29 ± 53.71	39.78 (8.82, 313.92)	60.42 ± 60.41	41.58 (8.82, 313.92)	50.55 ± 42.78	39.24 (17.1, 219.78)	0.372
GDR (mg/kg/min)	4.25 ± 1.81	3.88 (1.61, 8.91)	4.34 ± 1.76	4.13 (1.72, 8.91)	4.13 ± 1.89	3.64 (1.61, 8.58)	0.581
HOMA-IR	8.05 ± 7.98	5.36 (0.59, 46.37)	7.28 ± 8.23	5.11 (0.59, 46.37)	8.96 ± 7.79	6.99 (1.1, 40.84)	0.312
TyG index	8.12 ± 0.86	7.99 (6.38, 11.03)	8.13 ± 0.88	8.01 (6.38, 11.03)	8.11 ± 0.83	7.97 (6.94, 10.38)	0.897

T2DM, type 2 diabetes mellitus; HbA1c, glycosylated hemoglobin A1c; GDR, glucose disposal rate; HOMA-IR, homeostatic model assessment of insulin resistance; TyG index, triglyceride glucose index.

**Table 2:** Pearson’s correlations analysis between TyG index, HOMA-IR, and GDR.

	Men		Women		Total	
	Pearson’s Correlations Coefficient	*p* Value	Pearson’s Correlations Coefficient	*p* Value	Pearson’s Correlations Coefficient	*p* Value
TyG index and GDR	−0.412	0.001 **	−0.431	0.005 **	−0.418	0.0000 ****
HOMA-IR and GDR	−0.291	0.028 *	−0.364	0.019 *	−0.324	0.0011 **

* *p* < 0.05; ** *p* < 0.01; **** *p* < 0.0001.

**Table 3:** Multiple linear regression model for predicting IR.

	Men			Women		
	Additional R^2^	β	*p* Value	Additional R^2^	β	*p* Value
TyG index	0.134	−0.78	0.002 **	0.81	−0.69	0.02 *
HOMA-IR	0.044	−0.061	0.014 *	0.003	−0.034	0.369

The model was adjusted for age, duration of T2DM, metformin, BMI and waist circumference. * *p* < 0.05; ** *p* < 0.01.

**Table 4:** Odds ratios of multivariate logistic regression model for predicting IR.

	Men		Women	
	Odds ratio (95% Confidence Interval)	*p* Value	Odds ratio (95% Confidence Interval)	*p* Value
TyG index	2.22(1.12–4.39)	0.022 *	3.07(1.20–7.83)	0.019 *
HOMA-IR	1.08(1.00–1.15)	0.044 *	1.11(0.98–1.24)	0.092

The model was adjusted for age, duration of T2DM, metformin, BMI and waist circumference. * *p* < 0.05.

**Table 5:** Receiver operating characteristic (ROC) analysis for HOMA-IR and TyG index in the identification of insulin resistance.

	Men		Women		Total	
	AUC (95% Confidence Interval)	*p* Value	AUC (95% Confidence Interval)	*p* Value	AUC (95% Confidence Interval)	*p* Value
TyG index	0.774 (0.648–0.901)	0.02 *	0.794 (0.643–0.945)	0.015 *	0.785 (0.691–0.879)	0.001 **
HOMA-IR	0.709 (0.534–0.884)	0.076	0.769 (0.53–1.00)	0.027 *	0.73 (0.588–0.873)	0.006 **

* *p* < 0.05; ** *p* < 0.01.

## Data Availability

The data that support the findings of this study are available from Third Xiangya Hospital, Central South University, but restricted for research use only. The data are not publicly available. Data are available from the authors upon reasonable request and with permission of Third Xiangya Hospital, Central South University.
